# On the proportional hazards model for occupational and environmental case-control analyses

**DOI:** 10.1186/1471-2288-13-18

**Published:** 2013-02-15

**Authors:** Héloïse Gauvin, Aude Lacourt, Karen Leffondré

**Affiliations:** 1 Department of Social and Preventive Medicine, University of Montreal, PO Box 6128, Downtown Station, Montreal, Quebec H3C 3J7, Canada; 2 CHUM Research Centre, 3875 rue Saint-Urbain, Montreal, Quebec H2W 1V1, Canada; 3 University of Bordeaux, ISPED, Centre INSERM U897-Epidemiology-Biostatistics, 146 rue Leo Saignat, Bordeaux, F-33000, France

**Keywords:** Case-control study, Cox model, Logistic regression, Time-dependent variables, Variance estimator, Occupational exposures, Environmental exposures, Superpopulation

## Abstract

**Background:**

Case-control studies are generally designed to investigate the effect of exposures on the risk of a disease. Detailed information on past exposures is collected at the time of study. However, only the cumulated value of the exposure at the index date is usually used in logistic regression. A weighted Cox (WC) model has been proposed to estimate the effects of time-dependent exposures. The weights depend on the age conditional probabilities to develop the disease in the source population. While the WC model provided more accurate estimates of the effect of time-dependent covariates than standard logistic regression, the robust sandwich variance estimates were lower than the empirical variance, resulting in a low coverage probability of confidence intervals. The objectives of the present study were to investigate through simulations a new variance estimator and to compare the estimates from the WC model and standard logistic regression for estimating the effects of correlated temporal aspects of exposure with detailed information on exposure history.

**Method:**

We proposed a new variance estimator using a superpopulation approach, and compared its accuracy to the robust sandwich variance estimator. The full exposure histories of source populations were generated and case-control studies were simulated within each source population. Different models with selected time-dependent aspects of exposure such as intensity, duration, and time since cessation were considered. The performances of the WC model using the two variance estimators were compared to standard logistic regression. The results of the different models were finally compared for estimating the effects of correlated aspects of occupational exposure to asbestos on the risk of mesothelioma, using population-based case-control data.

**Results:**

The superpopulation variance estimator provided better estimates than the robust sandwich variance estimator and the WC model provided accurate estimates of the effects of correlated aspects of temporal patterns of exposure.

**Conclusion:**

The WC model with the superpopulation variance estimator provides an alternative analytical approach for estimating the effects of time-varying exposures with detailed history exposure information in case-control studies, especially if many subjects have time-varying exposure intensity over lifetime, and if only one control is available for each case.

## Background

Population-based case-control studies are widely used in epidemiology to investigate the association between environmental or occupational exposures over lifetime and the risk of cancer or other chronic diseases. Many of the exposures of interest are protracted and a huge amount of information is often retrospectively collected for each subject about his/her potential past exposure over lifetime. For example, for occupational exposures, the whole occupational history is usually investigated for each subject, and different methods exist to estimate the average dose of exposure at each past job [1-3]. However, only the cumulated estimated dose of exposure at the index age (age at diagnosis for cases and at interview for controls) is usually used in standard logistic regression analyses. Such approach does not use the (retrospective) dynamic information available on the exposure at different ages during lifetime.

A time-dependent weighted Cox (WC) model has recently been proposed to incorporate this dynamic information on exposure, in order to more accurately estimate the effect of time-dependent exposures in population-based case-control studies [4]. The WC model consists in using age as the time axis and weighting cases and controls according to their case-control status and the age conditional probabilities of developing the disease in the source population. The weights proposed in the WC model are therefore time-dependent and estimated from data of the source population. A simulation study showed that the WC model improved the accuracy of the regression parameters estimates of time-dependent exposure variables as compared with standard logistic regression with fixed-in-time covariates [4]. However, the average robust sandwich variance estimates based on dfbetas residuals [5] were systematically lower than the empirical variance of the parameter estimates, which resulted in too narrow confidence intervals (CI) and low coverage probabilities [4].

There is an extensive statistical literature on the weighted analyses of cohort sampling designs (see among many others [6-10]). A population-based case-control study can be seen as a nested case-control study within the source population of cases and controls, and can therefore fit in this general cohort sampling design framework. Population-based case-control studies can also be seen as a survey with complex selection probabilities [11-14] and this is the general framework that we use in this paper. Specifically, we consider the superpopulation approach developed by Lin [13] who proposed a variance estimator that accounts for the extra variation due to sampling the finite survey population from an infinite superpopulation. As a result, the Lin variance estimator accounts for the random variation from one survey sample to another and from one survey population to another, as opposed to the robust sandwich variance estimator that accounts only for the random variation from one survey sample to another. In the context of population-based case-control study, the case-control sample could be considered as the survey sample, the source population as the finite survey population, and the population under study as the infinite superpopulation.

The asymptotic properties of the Lin variance estimator have been investigated and a small simulation study has been conducted to investigate these properties in finite samples [13]. The results indicated that the superpopulation variance estimates were closer to the true variance than the robust sandwich variance estimates. However, the simulation study considered only fixed-in-time covariates and simple selection probabilities that did not reflect the more complex sampling scheme of population-based case-control studies. It is therefore unclear how the superpopulation variance estimator would perform for the estimation of the effects of time-dependent covariates using the specific estimated time-dependent weights proposed in the WC model [4]. In addition, for further applications to population-based case-control data, it would be important to clarify the performance of the WC model, as compared with standard logistic regression analyses, for estimating the effects of several correlated temporal patterns of protracted exposures. Indeed, the effects of temporal patterns of exposures such as intensity, duration, age at first exposure, and time since last exposure are often of great interest from an epidemiological point of view [15], but they need to be carefully adjusted for each other to avoid residual confounding [16]. Such adjustment induces correlation between covariates and it is important to investigate how it affects the proposed estimators.

The first objective of the present study is to investigate through extensive simulations the accuracy of the Lin variance estimator for estimating the effects of time-varying covariates in case-control data, using the weights proposed in the WC model [4]. The second objective is to compare the estimates from the WC model and standard logistic regression for estimating the effects of selected correlated temporal aspects of exposure with detailed information on exposure history. The next section introduces the WC model and the robust and Lin’s variance estimators. The different approaches are then compared through simulations and using data from a large population-based case-control study on occupational exposure to asbestos and pleural mesothelioma (PM).

## The regression model and the variance estimators

### The WC model

The Cox proportional hazards model specifies the hazard function as

$$\lambda \left(t|x\left(t\right)\right)=\lambda_{0}\left(t\right)\text{exp}\left\{x{\left(t\right)}^{\prime }\beta \right\},$$

where *λ*_0_ is the baseline hazard, *x*(*t*) is the vector of observed covariate values at time *t* and *β* is the vector of unknown regression parameters. In the context of a population-based survey with complex sampling design [5], the estimator of *β* is the solution of the pseudo-maximum likelihood equation

(1) $$U\left(\beta \right)=\sum_{i=1}^{n}\omega_{i}\delta_{i}\left\{x_{i}\left(t_{i}\right)-\frac{{\hat{S}}^{\left(1\right)}\left(t_{i},\hat{\beta }\right)}{{\hat{S}}^{\left(0\right)}\left(t_{i},\hat{\beta }\right)}\right\}=0,$$

where *n* is the sample size, *ω*_*i*_ is the sampling weight for subject *i*, *δ*_*i*_ = 1 if subject *i* is the case diagnosed at age *t*_*i*_ and 0 otherwise, and

$${\hat{S}}^{\left(0\right)}\left(t,\hat{\beta }\right)=\sum_{j=1}^{n}\omega_{j}Y_{j}\left(t\right)\text{exp}\left\{x_{j}{\left(t\right)}^{\prime }\hat{\beta }\right\},$$

$${\hat{S}}^{\left(1\right)}\left(t,\hat{\beta }\right)=\sum_{j=1}^{n}\omega_{j}Y_{j}\left(t\right)x_{j}\left(t\right)\text{exp}\left\{x_{j}{\left(t\right)}^{\prime }\hat{\beta }\right\},$$

with *Y*_*j*_(*t*) = 1 if the subject *j* is at risk at time *t* (i.e. *t*_*j*_ ≥ *t*), 0 otherwise.

In the WC model proposed for case-control data [4], *t* is age and the sampling weight *ω* of each subject depends on age and on his case-control status. Specifically, the weight for each subject *i* at age *t* is given by

(2) $$\omega_{i}\left(t\right)=\{\begin{array}{ll} \frac{1-\pi \left(t\right)}{\pi \left(t\right)}\times \frac{n_{\text{cases}}\left(t\right)}{n_{\text{controls}}\left(t\right)} & \mathrm{if}\;\text{subject}\;i\;\mathrm{is}\;\text{a}\;\text{control}\;\text{selected}\;\mathrm{at}\;\text{age}\;t\;\mathrm{or}\;\mathrm{at}\;\text{a}\;\text{later}\;\text{age}\\ 1 & \mathrm{if}\;\text{subject}\;i\;\mathrm{is}\;\text{a}\;\text{case}\;\text{diagnosed}\;\mathrm{at}\;\text{age}\;t\;\mathrm{or}\;\mathrm{at}\;\text{a}\;\text{later}\;\text{age}, \end{array}$$

where *π*(*t*) is the probability to develop the disease at age *t* or at a later age in the source population, *n*_*cases*_(*t*) is the number of cases diagnosed at age *t* or at a later age in the case-control study, and *n*_*controls*_(*t*) is the number of controls selected at age *t* or at a later age in the case-control study as well. If the WC model is used to analyze data from a nested case-control study, the age conditional probabilities *π*(*t*) in Equation (2) can directly be estimated from the full enumerated cohort. Left-truncation at age at entry into the cohort should be performed to account for delayed entry [17]. If the WC model is used to analyze population-based case-control data, *π*(*t*) can be estimated from health statistics on the population under study, as shown in our application on PM in the section following simulations. The weights equal 1 for cases because all the eligible cases of the source population (or in the cohort) are usually included in the case-control study. If the sampling probabilities of cases do not equal 1, then weights in Equation (2) should be adjusted accordingly.

The weights defined in Equation (2) can be implemented in any statistical software that handles time-dependent weights in the Cox model, such as the coxph function in R or the SAS PROC PHREG function.

### The variance estimators

The robust sandwich variance estimator for $\hat{\beta }$ in Equation (1) as proposed by Binder [5] for finite population-based surveys is given by

(3) $${\hat{V}}_{1}\left(\hat{\beta }\right)=I^{-1}\left(\hat{\beta }\right)\left[\sum_{i=1}^{n}{\left\{\omega_{i}{\hat{u}}_{i}\left(\hat{\beta }\right)\right\}}^{⊗2}\right]I^{-1}\left(\hat{\beta }\right)$$

where $I\left(\hat{\beta }\right)$ is the observed information matrix obtained by evaluation of this expression ${\frac{\partial \hat{U}\left(\beta \right)}{\partial \beta }|}_{\beta =\hat{\beta }}$, *a*^⊗ 2^ = *aa*′, and

(4) $$\begin{array}{l} {\hat{u}}_{i}\left(\hat{\beta }\right)=\delta_{i}\left\{x_{i}\left(t_{i}\right)-\frac{{\hat{S}}^{\left(1\right)}\left(t_{i},\hat{\beta }\right)}{S^{\left(0\right)}\left(t_{i},\hat{\beta }\right)}\right\}\\ \;-\sum_{j=1}^{n}\delta_{j}\omega_{j}\frac{Y_{i}\left(t_{j}\right)\text{exp}\left\{x_{i}{\left(t_{j}\right)}^{\prime }\hat{\beta }\right\}}{{\hat{S}}^{\left(0\right)}\left(t_{j},\hat{\beta }\right)}\\ \;x\;\left\{x_{i}\left(t_{j}\right)-\frac{{\hat{S}}^{\left(1\right)}\left(t_{j},\hat{\beta }\right)}{{\hat{S}}^{\left(0\right)}\left(t_{j},\hat{\beta }\right)}\right\}. \end{array}$$

The robust variance estimator in Equation (3) can be rewritten as ${\hat{V}}_{1}\left(\hat{\beta }\right)$*=D’D* where *D* is the dfbetas residuals [18] vector from the Cox model including the weights *ω* that can depend on time as those defined in Equation (2), as suggested in Barlow [19]. As indicated by Therneau and Li [20] and by Barlow et al. [21], the robust sandwich variance estimate from Equation (3) can directly be obtained with R using the commands

M1 < −coxph(Surv(start,stop,event) ~ x + cluster(id), weights = weight)

V1 < − M1$var

with the vector of weights derived from Equation (2) for the WC model.

The robust variance estimator ${\hat{V}}_{1}\left(\hat{\beta }\right)$ accounts for the variability due to sampling the case-control sample from the source population. To account for the extra variability due to sampling the source population from the (infinite) superpopulation, we propose to use the Lin variance estimator [13] that turned out to consist in adding the naïve variance estimator to the robust variance estimator ${\hat{V}}_{1}\left(\hat{\beta }\right)$. The Lin variance superpopulation estimator is thus given by

(5) $${\hat{V}}_{2}\left(\hat{\beta }\right)={\hat{V}}_{1}\left(\hat{\beta }\right)+I^{-1}\left(\hat{\beta }\right).$$

With R, the superpopulation variance estimate from Equation (5) can simply be obtained using the command

V2 <- V1 + M1$naive.var

All along this paper, the WC model using the robust variance estimator ${\hat{V}}_{1}\left(\hat{\beta }\right)$ in Equation (3) will be denoted by WC1, while the WC model using the Lin’s superpopulation variance estimator ${\hat{V}}_{2}\left(\hat{\beta }\right)$ in Equation (5) will be denoted by WC2. While WC1 and WC2 models give identical estimated exposure effects, they yield different standard errors and thus CI.

## Simulations

### Overview of the simulation design

The main objective of the simulation study was to evaluate the performance of Lin’s superpopulation variance estimator ${\hat{V}}_{2}\left(\hat{\beta }\right)$ in Equation (5) with the time-dependent weights defined in Equation (2), for the estimation of the effects of time-varying exposures in case-control studies. In particular, we compared the coverage probability of the 95% CI resulting from the WC2 model, as compared to the WC1 model and standard logistic regression. We were specifically interested in the effects of exposure intensity, duration, age at first exposure and time since last exposure. These inter-related aspects of exposure are of interest in many epidemiological applications but induce some statistical analytical issues because of correlation and time-dependency.

We generated 1000 source populations of 1000 or 5000 individuals each, and within each source population, we simulated a case-control study. The age at event for each subject in each source population was generated from a standard Cox model with time-dependent covariates, using a permutation algorithm described elsewhere and assuming Weibull marginal distribution of age at event [4,22,23]. Three Cox models of interest from an epidemiological point of view were simulated. Model 1 included intensity and duration of exposure only. Model 2 included age at first exposure in addition to intensity and duration. Model 3 was similar to Model 2 but used time since last exposure instead of age at first exposure.

The distribution of the exposures variables were chosen to be close to the observed distributions of occupational asbestos exposure variables in our case-control data on PM [15] described in the application section. Specifically, the ages at first and at last exposure were generated for all subjects from lognormal distributions. The exposure intensity at each age was generated from a linear function of age. Parameters for the random intercept and slope were chosen such that either 85% of subjects had a constant intensity, 6% a highly increasing, 6% a moderately decreasing, and 3% a moderately increasing intensity over lifetime (Scenario A); or 50% a highly increasing and 50% a moderately decreasing intensity over lifetime (Scenario B). Scenario A reflects our real case-control data on occupational exposure to asbestos. The exposure intensity at each age was represented in all our models by a variable that equaled the cumulated value of intensity at that age divided by the total duration of exposure at that age. This exposure intensity variable is equivalent to the mean index of exposure (MIE) variable introduced in the application section. The exposure intensity, as well as duration and time since last exposure, were time-dependent in all our true Models 1–3. The true effects *β* of each exposure variable in Models 1–3 were fixed to values that ranged from weak to strong effects: 0.41 to 1.39 for intensity, 0.01 to 0.05 for duration, −0.01 to −0.11 for age at first exposure, and 0.01 to 0.04 for time since last exposure. These beta correspond to hazard ratios of 1.5 to 4.0 for one standard deviation (i.e. 1.0 fiber/ml) increase in exposure intensity, hazard ratios of 1.2 to 2.0 for one standard deviation increase (i.e. 14 years) in duration of exposure, hazard ratios of 0.9 to 0.4 for one standard deviation (i.e. 8 years) increase in age at first exposure, and hazard ratios of 1.2 to 1.8 for one standard deviation (i.e. 14 years) increase in time since cessation of exposure.

Censoring for age at event in the source population was independently generated from a uniform distribution such that the event rate was about 10% in each source population of 1000 subjects, and 2% in each source population of 5 000 subjects. Each subject of the source population who had the event of interest was selected as a case in the case-control dataset. The event rates in the source population thus implied that we had about 100 cases in each case-control data set. For each case, 1, 2, or 4 controls were randomly selected with replacement among subjects at risk at the case’s event age, which corresponds to 1:1, 1:2, or 1:4 individual matching on age, respectively. On average, each case-control dataset was therefore made of about 100 cases and 100, 200, or 400 controls.

## Analytical methods used to analyze the simulated data

Each case-control sample was analyzed using four regression models (WC1 and WC2 models and two standard logistic regression models) that were correctly specified in terms of the exposure variables included. In the WC1 and WC2 models, the exposure variables were time-dependent, and the probability *π*(*t*) was the proportion of subjects in the source population who had an event at age *t* or at a later age among those at risk at age *t*. We assumed that all subjects of the population source were followed-up since birth, implying that age at event did not have to be left-truncated in WC1 and WC2. For comparison purpose, conditional logistic regression (CLR) was used as the standard analytical method for individually matched case-control studies. Unconditional logistic regression (ULR) including age as a continuous covariate in addition to the exposure variables, was also used as the standard alternative analytical approach. For both ULR and CLR, the time-dependent covariates were fixed at their observed value at the age at event for cases or selection for controls. Because controls were selected among subjects at risk at the ages where each case occurs, all the exponential of the regression parameter estimates can be interpreted as the source population rate ratio estimates [24].

### Statistical criteria used to compare the performance of the different estimators

For each of the four regression models WC1, WC2, CLR, and ULR, we calculated the relative bias of the regression parameter estimator $\hat{\beta }$ associated with each exposure variable, as compared with the true effect *β* of that exposure variable, $\frac{1}{1000}\sum_{i=1}^{1000}\left(\frac{{\hat{\beta }}_{i}-\beta }{\beta }\right)$, where ${\hat{\beta }}_{i}$ is the parameter estimate of the model based on the *i*^th^ case-control dataset (i = 1, …, 1 000). To evaluate whether the relative bias was not partly due to a bias generated in the population source data, we also derived the relative bias as compared with the estimated effect ${\hat{\beta }}_{\text{Cox}}$ of the well specified time-dependent Cox model using the full population source data, $\frac{1}{1000}\sum_{i=1}^{1000}\left(\frac{{\hat{\beta }}_{i}-{\hat{\beta }}_{\text{Cox},i}}{{\hat{\beta }}_{\text{Cox},i}}\right)$. We also derived the root mean squared error (RMSE) $\sqrt{{\left(\bar{\hat{\beta }}-\beta \right)}^{2}+\text{var}\left(\hat{\beta }\right)}$, where $\bar{\hat{\beta }}$ is the mean of the 1 000 parameter estimates ${\hat{\beta }}_{i}$. The empirical relative efficiency of each regression parameter estimator was computed as the ratio of the empirical variance of the Cox model using the full source population data, $\text{var}\left({\hat{\beta }}_{\text{Cox}}\right)$, to the empirical variance of the parameter estimates $\text{var}\left(\hat{\beta }\right)$. The average of the 1000 standard errors $s\left(\hat{\beta }\right)$ (ASE) was compared to the empirical standard deviation of the 1000 $\hat{\beta }$ estimates (SDE). We also calculated the coverage probability as the proportion of samples for which the 95% CI of *β,*$\hat{\beta }\pm 1.96\times s\left(\hat{\beta }\right)$, included the true value *β*.

### Simulation results

Table 1 shows the results of the four analytical methods (WC1, WC2, CLR, ULR) for strong effects of exposure intensity and duration in Model 1. Table 2 shows the results for strong effects of i) intensity, duration, and age at first exposure in Model 2, and ii) intensity, duration, and time since cessation in Model 3. The results tended to be similar for weaker effects.

**Table 1:** Simulation results for Model 1 for 1:1, 1:2, or 1:4 matched case-control data including about 100 cases arising from populations of 1000 or 5000 subjects, based on 1000 replications

**Population size**	**Case: control ratio**	**Intensity patterns (a)**	**Exposure variables**	***β***	**Method (b)**	**Relative bias (%) (c)**	**Relative bias / Cox pop (%) (c)**	**Relative efficiency (d)**	**RMSE × 10**^**-3**^**(e)**	**ASE/SDE (e)**	**Cov. rate (e)**
1 000	1:1	A	Intensity	1.39	WC1	2.9	2.4	0.61	158	0.87	89.1
					WC2	-	-	-	-	1.17	97.5
					CLR	5.9	5.5	0.14	327	0.95	96.5
					ULR	−2.6	−3.0	0.31	218	0.97	93.1
			Duration	0.05	WC1	3.3	2.0	0.41	14	0.82	88.3
					WC2	-	-	-	-	1.08	97.1
					CLR	6.2	4.6	0.19	20	0.96	95.7
					ULR	−5.3	−6.6	0.35	15	1.03	95.1
	1:1	B	Intensity	1.39	WC1	2.6	2.7	0.59	158	0.88	89.9
					WC2	-	-	-	-	1.18	98.7
					CLR	3.4	3.4	0.14	315	0.94	94.9
					ULR	−3.8	−3.7	0.31	219	0.98	92.0
			Duration	0.05	WC1	2.0	2.4	0.45	14	0.79	88.3
					WC2	-	-	-	-	1.04	96.1
					CLR	1.9	2.2	0.21	21	0.94	94.2
					ULR	−8.6	−8.3	0.39	16	0.99	93.4
5 000	1:1	B	Intensity	1.39	WC1	7.3	9.3	0.20	254	0.72	76.1
					WC2	-	-	-	-	0.85	85.6
					CLR	−0.9	0.7	0.10	325	0.87	89.8
					ULR	−3.3	−1.7	0.23	219	0.92	91.8
			Duration	0.05	WC1	1.6	7.0	0.17	28	0.79	89.0
					WC2	-	-	-	-	0.90	93.0
					CLR	−15.7	−12.5	0.19	27	0.92	90.8
					ULR	−15.4	−11.9	0.32	22	0.94	90.4
	1:2	B	Intensity	1.39	WC1	−0.3	1.6	0.25	203	0.78	86.7
					WC2	-	-	-	-	0.93	92.8
					CLR	−3.0	−1.2	0.22	218	0.98	93.1
					ULR	−3.5	−1.7	0.34	181	0.96	91.8
			Duration	0.05	WC1	−3.4	1.3	0.27	22	0.85	89.2
					WC2	-	-	-	-	1.00	94.5
					CLR	−10.0	−6.5	0.33	21	0.95	92.3
					ULR	−10.2	−6.5	0.45	18	0.96	93.3
	1:4	B	Intensity	1.39	WC1	−5.3	−3.6	0.37	191	0.80	85.0
					WC2	-	-	-	-	0.99	91.5
					CLR	−3.9	−2.2	0.36	187	0.93	89.8
					ULR	−3.6	−1.8	0.47	164	0.93	91.0
			Duration	0.05	WC1	−10.6	−6.5	0.39	19	0.86	88.9
					WC2	-	-	-	-	1.06	94.6
					CLR	−11.1	−7.3	0.49	17	0.95	91.7
					ULR	−10.9	−6.9	0.58	16	0.95	92.6

(a) Exposure intensity was either constant over lifetime for 85% of the subjects, highly increasing for 6%, moderately decreasing for 6%, and moderately increasing intensity for 3% (Scenario A); or, was highly increasing for 50% and moderately decreasing for 50% (Scenario B).

(b) WC1, weighted Cox models with robust sandwich variance; WC2, weighted Cox model with superpopulation variance; CLR, conditional logistic regression on age; ULR, unconditional logistic regression adjusted for age as a continuous covariate.

(c) Relative bias as compared to the true effect and as compared to the estimated effect of the Cox model using the full population source data. Each of these two bias was the same for WC1 and WC2 since these models used the same regression parameter estimator $\hat{\beta }$.

(d) Relative efficiency as compared to the Cox model estimated on the full population source. This quantity was the same for WC1 and WC2 since these models used the same regression parameter estimator $\hat{\beta }$.

(e) RMSE, root mean squared error (same for WC1 and WC2 which used the same regression parameter estimator $\hat{\beta }$); ASE, average of the 1000 standard errors $s\left(\hat{\beta }\right)$; SDE, empirical standard deviation of the 1000 $\hat{\beta }$ estimates; cov. rate, coverage rate of the 95% confidence interval of $\hat{\beta }$.

**Table 2:** Simulation results for Models 2 and 3 for 1:1 matched case-control data including about 100 cases arising from a population of 1000 subjects, based on 1000 replications

**Model**	**Intensity patterns (a)**	**Exposure variables**	***β***	**Method (b)**	**Relative bias (%) (c)**	**Relative bias / Cox pop (%) (c)**	**Relative efficiency (d)**	**RMSE × 10**^**-3**^**(e)**	**ASE/SDE (e)**	**Cov. rate (e)**
2	A	Intensity	1.39	WC1	3.7	2.7	0.60	164	0.86	91.1
				WC2	-	-	-	-	1.18	98.3
				CLR	9.5	8.3	0.09	435	0.82	96.3
				ULR	−2.9	−3.9	0.28	230	0.97	94.1
		Duration	0.05	WC1	1.5	1.9	0.44	16	0.80	88.5
				WC2	-	-	-	-	1.05	95.7
				CLR	4.9	4.7	0.13	29	0.84	95.5
				ULR	−11.9	−11.8	0.36	19	1.01	93.6
		Age at first exposure	−0.11	WC1	4.7	3.1	0.44	32	0.79	86.3
				WC2	-	-	-	-	1.04	95.3
				CLR	9.9	7.7	0.18	50	0.92	95.1
				ULR	0.4	−1.2	0.39	33	1.00	95.1
	B	Intensity	1.39	WC1	3.1	2.8	0.64	161	0.88	90.1
				WC2	-	-	-	-	1.19	98.4
				CLR	6.9	6.5	0.10	405	0.84	94.1
				ULR	−4.4	−4.7	0.32	229	0.98	93.3
		Duration	0.05	WC1	1.3	1.3	0.49	16	0.82	90.4
				WC2	-	-	-	-	1.09	96.5
				CLR	5.6	5.0	0.17	27	0.91	95.0
				ULR	−12.7	−12.8	0.37	19	1.00	92.9
		Age at first exposure	−0.11	WC1	3.6	2.8	0.51	30	0.83	89.8
				WC2	-	-	-	-	1.09	96.5
				CLR	7.7	6.1	0.17	53	0.87	94.7
				ULR	−1.7	−2.6	0.40	34	0.99	95.5
3	A	Intensity	1.39	WC1	3.4	3.0	0.58	165	0.84	90.3
				WC2	-	-	-	-	1.13	97.0
				CLR	6.0	5.5	0.14	333	0.92	95.9
				ULR	−1.5	−1.9	0.33	213	0.99	94.2
		Duration	0.05	WC1	0.3	0.0	0.47	23	0.80	88.7
				WC2	-	-	-	-	1.05	96.1
				CLR	5.2	5.3	0.24	32	0.93	95.1
				ULR	−2.7	−2.7	0.40	24	0.98	95.1
		Time since cessation	0.04	WC1	0.8	2.3	0.43	27	0.78	87.3
				WC2	-	-	-	-	1.02	95.9
				CLR	8.0	4.2	0.24	36	0.93	95.4
				ULR	2.9	−6.0	0.38	28	0.97	95.2
	B	Intensity	1.39	WC1	2.9	3.0	0.63	160	0.88	90.4
				WC2	-	-	-	-	1.18	98.8
				CLR	4.6	4.6	0.15	326	0.92	95.9
				ULR	−2.8	−2.7	0.36	208	1.02	93.7
		Duration	0.05	WC1	−0.7	1.1	0.44	23	0.79	86.9
				WC2	-	-	-	-	1.04	95.9
				CLR	−1.8	0.6	0.24	31	0.94	95.4
				ULR	−7.7	−6.2	0.39	25	0.98	94.5
		Time since cessation	0.04	WC1	−1.2	11.2	0.46	26	0.82	88.7
				WC2	-	-	-	-	1.07	95.4
				CLR	−0.3	9.5	0.25	35	0.97	95.6
				ULR	−2.3	−13.2	0.40	27	1.01	95.6

(a) Exposure intensity was either constant over lifetime for 85% of the subjects, highly increasing for 6%, moderately decreasing for 6%, and moderately increasing intensity for 3% (Scenario A); or, was highly increasing for 50% and moderately decreasing for 50% (Scenario B).

(b) WC1, weighted Cox models with robust sandwich variance; WC2, weighted Cox model with superpopulation variance; CLR, conditional logistic regression on age; ULR, unconditional logistic regression adjusted for age as a continuous covariate.

(c) Relative bias as compared to the true effect and as compared to the estimated effect of the Cox model using the full population source data. Each of these two bias was the same for WC1 and WC2 since these models used the same regression parameter estimator $\hat{\beta }$.

(d) Relative efficiency as compared to the Cox model estimated on the full population source. This quantity was the same for WC1 and WC2 since these models used the same regression parameter estimator $\hat{\beta }$.

(e) RMSE, root mean squared error (same for WC1 and WC2 which used the same regression parameter estimator $\hat{\beta }$); ASE, average of the 1000 standard errors $s\left(\hat{\beta }\right)$; SDE, empirical standard deviation of the 1000 $\hat{\beta }$ estimates; cov. rate, coverage rate of the 95% confidence interval of $\hat{\beta }$.

As suggested by the ratio ASE/SDE, the superpopulation variance estimator (WC2) tended to give estimates that were closer to the true variance than the robust variance estimator (WC1) that systematically under-estimated the true variance. Despite the superpopulation variance estimator tended to overestimate the true variance for the effect of exposure intensity when the population was made of 1000 subjects only (Tables 1 and 2), the coverage rates from WC2 were systematically much closer to the nominal level of 95% than those from the WC1 model. For each scenario of intensity pattern (Scenario A or B), the ratio ASE/SDE and the coverage rate for the effects of intensity and duration were similar in Models 2–3 as compared with Model 1 (Table 2 versus Table 1), suggesting that additional adjustment for correlated covariates does not affect the performance of the different variance estimators.

While the relative biases from all analytical models (WC, ULR and CLR) tended to be low and of the same magnitude in all scenarios, the relative efficiency as compared to the Cox model estimated on the full population source, as well as the accuracy in terms of RMSE, tended to be different. Indeed, in all scenarios with 1:1 case:control ratio within population source of 1000 subjects, the regression coefficient estimator from the WC models was much more efficient and thus also more accurate than that from CLR and ULR (Tables 1 and 2). As expected, the relative efficiency from all models estimated using 100 cases and 100 controls, as compared to the Cox model estimated on the full population source, decreased when the population size increases. For example, the relative efficiency of the WC for intensity with pattern B decreased from 0.59 to 0.20 when the population size increased from 1000 to 5000 subjects (Table 1). As expected as well, increasing the number of controls from 100 to 200 or 400 for a given population size (5000 in Table 1) strongly increased the relative efficiency of ULR and CLR but only moderately increased the relative efficiency of the WC models. For example, the relative efficiency for intensity with pattern B increased from 0.10 to 0.36 for CLR while only from 0.20 to 0.37 for the WC model (Table 1). Because the WC model used controls at different ages for which they were selected in the 1:1 case-control scenario, using additional controls in the 1:2 or 1:4 case:control ratio scenarios added relatively less information in this model than in ULR and CLR. As a result, ULR and CLR became more accurate in terms of RMSE than the WC models when four controls were selected for each case.

Interestingly, CLR did not perform better in terms of both bias and RMSE than ULR, despite individual matching of cases and controls. ULR was actually systematically more efficient than CLR. This result may be consistent with our previous results where we found that CLR might have difficulty in separating the effects of correlated time-dependent variables [23]. Indeed, the correlation between each pair of the four exposure variables (intensity, duration, age at first exposure and time since last exposure) as well as with age at the index date, ranged between −0.679 and +0.453. The correlation also affected both the WC and ULR parameter estimators as suggested by the slightly higher RMSE in Models 2 and 3 (Table 2) as compared with Model 1 (Table 1) for the effects of intensity and duration, but it affected them less than the CLR estimator.

### Application to occupational exposure to asbestos and pleural mesothelioma

Mesothelioma is a rare tumor mostly located in the pleura and usually caused by exposure to asbestos. The role of the different temporal patterns of occupational exposure to this substance has still to be explored using appropriate statistical methods accounting for individual changes over time in the exposure intensity [15]. It is therefore of interest to apply the proposed estimators to estimate the mutually adjusted effects of exposure intensity, duration, age at first exposure, and time since last exposure, and to compare the results to those from standard logistic regression analyses that do not dynamically account for within subjects changes over time of exposure intensity.

### Data source

The data came from a large French population-based case-control study described in Lacourt et al. [15]. Cases were selected from a French case-control study conducted in 1987–1993 and the French National Mesothelioma Surveillance Program in 1998–2006. Population controls were frequency matched to cases by sex and year of birth within 5 years group. Occupational asbestos exposure was evaluated for each subject with a job-exposure matrix (JEM) which allowed us to derive the mean index of exposure (MIE) that was used in the regression models to represent intensity of exposure, as in Lacourt et al. [15]. The MIE at age *t* was given by

$$\text{MIE}\left(t\right)=\left(\sum_{l=1}^{L}d_{l}\times p_{l}\times \left[\left(f_{\mathrm{sl}}\times i_{\mathrm{sl}}\right)+\left(f_{\mathrm{al}}\times i_{\mathrm{al}}\right)\right]\right)/\sum_{l=1}^{L}d_{l}$$

where *L* is the total number of jobs exposed to asbestos till age *t*; *d*_*l*_ the duration (in years) of job *l*, *p*_*l*_ the probability of asbestos exposure for job *l*, *f*_s*l*_ and *i*_s*l*_ the frequency and intensity of asbestos exposure due to specific task of job *l*, respectively, *f*_a*l*_ and *i*_a*l*_ the frequency and intensity of asbestos exposure due to environment work contamination of job *l*, respectively. For each job, the probability was derived from the percent of workers exposed in the considered job code, the frequency from the percent of work time, and the intensity from the concentration of asbestos fibers in the air expressed as fibers per milliliter (f/ml). See Lacourt et al. [15] for more details. An ever exposed subject to asbestos was a subject who had at least one job with a probability *p*_*l*_ different from zero.

Because our objective was to accurately investigate the effects of the quantitative time-related aspects of occupational exposure, all our analyses were restricted to subjects ever exposed to asbestos (68.9% in males and 20.9% in females). In addition, because the sample size for females was too small to ensure adequate statistical power and accurate estimates in separate multiple regression analyses of this group [15], the analyses were restricted to males ever exposed to asbestos, i.e. to 1041 male cases and 1425 male controls. The distribution of age and the asbestos exposure characteristics at the time of diagnosis for cases and interview for controls are shown in Table 3. The distribution of the patterns of intensity over lifetime was similar to the one described in scenario A of the simulation, with 85% of subjects with almost constant asbestos exposure intensity over lifetime.

**Table 3:** Mean and standard deviation of age and asbestos exposure variables at the time of diagnosis/interview for ever exposed males

**Characteristics**	**Cases**	**Controls**
	**(*n*** **= 1 041)**	**(*n*** **= 1 425)**
Age at diagnosis / interview (years)	67.0 (10.0)	65.9 (6.3)
Year of birth	1 931.1 (10.0)	1 931.0 (9.3)
Age at first exposure (years)	21.0 (7.1)	22.6 (8.1)
Mean exposure intensity over lifetime (fibers/ml) (a)	0.62 (1.43)	0.21 (0.44)
Total exposure duration (years)	27.8 (12.9)	25.0 (14.1)
Time since last exposure (years)	16.9 (13.4)	17.4 (14.6)

Results from the French case-control study on mesothelioma, 1987–2006.

(a) Measured by the mean index of exposure (MIE).

### Analytical methods used to analyze the case-control data on pleural mesothelioma

To derive the weights proposed in the WC models (Equation 2), we first estimated the age-conditional probabilities *π*(*t*) of developing PM in the French male general population. These estimated probabilities were derived from published estimated sex- and age-specific incidence rates of PM per 100000 person-years in France in 2005 [25]. We assumed that these estimated incidence rates applied to our source population and that they were appropriate during the whole life of our subjects. The results are shown in Table 4 for males. As in the simulation study, standard errors for the WC model were then derived using the two variance estimators ${\hat{V}}_{1}\left(\hat{\beta }\right)$ and ${\hat{V}}_{2}\left(\hat{\beta }\right)$, resulting in the WC1 and WC2 models, respectively.

**Table 4:** Estimated male age-conditional probabilities used in the weights of the WC models to analyze the French case-control study of on mesothelioma

**Age *t***	***p*(*t*) (a)**	***π*(*t*) (b)**
0-44	0.1	0.000942
45-49	0.4	0.000941
50-54	1.2	0.000937
55-59	2.8	0.000925
60-64	5.2	0.000897
65-69	8.0	0.000845
70-74	10.5	0.000765
75-79	13.2	0.000660
80-84	15.2	0.000528
85-89	14.5	0.000376
90-94	11.6	0.000231
95 or more	11.5	0.000115

(a) *p*(*t*) are estimated male age-specific incidence rates of pleural mesothelioma per 100 000 person-years in France in 2005 [25].

(b) *π*(*t*) are estimated male age-conditional probabilities of developing pleural mesothelioma within residual lifetime after age *t*, calculated as $\pi \left(t\right)=1-\prod_{l\geq t}\left(1-p\left(l\right)\right)$.

For comparison purpose, the data were further analyzed with ULR which is the standard method to analyze frequency matched case-control data, as well as with CLR. Age was the time axis in WC1 and WC2 models, and a continuous covariate in ULR and CLR. We did not perform left-truncation in WC1 and WC2 models thus assuming that all subjects of the population source were passively followed-up for PM since birth. The matching factor, birth year, was a quantitative covariate in WC1, WC2, and ULR, and was the stratification variable (in 5 years groups) in CLR. Using each of the four approaches (WC1, WC2, CLR and, ULR), we estimated the effects of intensity and duration of occupational asbestos exposure, the age at first exposure, and time since last exposure, using the same combination of quantitative exposure variables as in Models 1–3 of the simulation study. All the effects of these variables were therefore assumed to be linear. Despite our recent results that suggested that these effects were not linear on the logit of PM [15], we used quantitative variables in order to facilitate the comparison of the estimates from the four different analytical approaches. The resulting estimates should therefore be used only for methodological comparison purpose and not as substantive epidemiological results. As in the simulation study, all the exposure variables were time-dependent in WC1 and WC2 models, and fixed at their value at the age at diagnosis or interview for ULR and CLR.

## Results

Table 5 shows the estimated effects of the selected quantitative asbestos exposure variables on the risk of PM, using the four analytical approaches (WC1, WC2, CLR, and ULR) and Models 1–3. The estimated effects are shown in terms of $\text{exp}\left(\hat{\beta }\right)$, i.e. estimated hazard ratios for WC1 and WC2 and estimated odds ratios for ULR and CLR. These estimated effects were calculated for an increase of about one standard deviation of the exposure variable, i.e. 1 fiber/ml for asbestos exposure intensity, 14 years for duration, 8 years for age at first exposure, and 14 years for time since last exposure.

**Table 5:** Estimated effect of occupational asbestos exposure in males ever exposed (1041 cases and 1425 controls), using the WC models and logistic regression and assuming linear effects of quantitative exposure variables

**Model**	**Exposure variables (a)**	**Unit**	**Method (b)**	$\text{exp}\left(\hat{\beta }\right)$**(c)**	**95% CI**	
1	Intensity	1.0 fiber/ml	WC1	1.75	1.66	1.84
			WC2	-	1.65	1.85
			CLR	2.55	2.29	2.83
			ULR	2.33	2.14	2.54
	Duration	14 years	WC1	1.32	1.24	1.40
			WC2	-	1.23	1.41
			CLR	1.18	1.12	1.24
			ULR	1.17	1.12	1.23
2	Intensity	1.0 fiber/ml	WC1	1.73	1.64	1.82
			WC2	-	1.63	1.83
			CLR	2.49	2.24	2.76
			ULR	2.31	2.12	2.52
	Duration	14 years	WC1	1.19	1.12	1.27
			WC2	-	1.11	1.28
			CLR	1.08	1.02	1.14
			ULR	1.10	1.05	1.15
	Age at first exposure	8 years	WC1	0.63	0.58	0.68
			WC2	-	0.57	0.70
			CLR	0.66	0.61	0.72
			ULR	0.77	0.73	0.82
3	Intensity	1.0 fiber/ml	WC1	1.74	1.65	1.83
			WC2	-	1.64	1.84
			CLR	2.53	2.28	2.82
			ULR	2.33	2.14	2.53
	Duration	14 years	WC1	1.90	1.68	2.14
			WC2	-	1.64	2.19
			CLR	1.41	1.27	1.57
			ULR	1.41	1.29	1.53
	Time since last exposure	14 years	WC1	1.55	1.37	1.75
			WC2	-	1.34	1.79
			CLR	1.24	1.11	1.39
			ULR	1.25	1.14	1.37

Results from the French case-control study on mesothelioma, 1987–2006.

(a) All the exposure variables were time-dependent in WC1 and WC2 models, and fixed at their value at diagnosis/interview in CLR and ULR. Intensity was measured by the mean index of exposure (MIE).

(b) WC1, weighted Cox models with robust sandwich variance; WC2, weighted Cox model with superpopulation variance; Both WC1 and WC2 used age as the time axis and included birth year as a quantitative covariate; ULR, unconditional logistic regression including age at diagnosis/interview and birth year as quantitative covariates; CLR, conditional logistic regression stratified on birth year group (5 years), and including age at diagnosis/interview as a quantitative covariate.

(c) Hazard ratio estimates for WC1 and WC2 (same value for WC1 and WC2) and odds ratio estimates for CLR and ULR, adjusted for age and birth year, and corresponding 95% confidence interval (CI).

As expected, the associations between all asbestos exposure variables and PM were significant with each of the four analytical approaches (Table 5). Specifically, increasing intensity or duration increased significantly the risk of PM, when adjusted or not on either age at first exposure or time since last exposure. Because the relative variation in the estimated effects of duration between Model 3 and Model 1 was higher than between Model 2 and Model 1, time since last exposure (in Model 3) seems to be a more important confounder than age at first exposure (in Model 2) in the relation between duration and PM. Estimates from Model 2 suggest that the later a subject is first occupationally exposed to asbestos, the smaller his risk of PM is. All the estimated effects of time since cessation indicate that risk continues to increase after the cessation of exposure, as in many other studies [15,26,27].

The 95% CI from WC1 and WC2 were almost identical (Table 5), suggesting that the robust variance estimates from WC1 was very close to the superpopulation variance estimates from WC2. This is likely due to the fact that the disease (PM) was very rare as shown in Table 3, as opposed to our simulation study where the overall event rates were about 10% and 2%.

The strongest contrasts between the estimates from the WC models and ULR or CLR were for the effect of exposure intensity. Indeed, the estimated effect of intensity was systematically weaker with the WC models than with ULR or CLR, with even non overlapping 95% CI. Note that, as for Scenario A in our simulation study, CLR provided the strongest estimates for the strong effect of intensity. By contrast, for the effects of duration, age at initiation, and time since last exposure, the strongest estimates were provided by the WC models, but the discrepancies with ULR and CLR were weaker than for intensity.

There are different potential explanations for the discrepancies between the results from the Cox (WC1 and WC2) and logistic (CLR and ULR) models. First the adjustment for age was largely different in the two series of models. While age was the time axis in the Cox models, and was therefore adequately adjusted for in both WC1 and WC2, it was included as a continuous covariate in both logistic models. This assumed that its effect was linear on the logit, which is actually not true [15]. Thus there may be some residual confounding by age in both CLR and ULR. Second, because controls of the case-control study on PM were selected from members of the general French population at calendar times that can possibly differ from the period of case’s recruitment, the case-control odds ratio estimate from ULR and CLR may estimate a different quantity than the hazard ratio estimate from the Cox model. Indeed, the hazard function in the Cox models provides a dynamic description of how the instantaneous risk of getting PM varies over the age. The exponential of regression parameter can be interpreted as a hazard ratio, which is equivalent to the rate ratio that would be obtained from a cohort design. If the controls of the case-control study on PM were randomly selected from the member of the population who were at risk at each age a case occurs (as in our simulation study), then the estimated odds ratio that would be obtained from ULR and CLR could also be interpreted as a rate ratio that would be obtained from a cohort design. However, this was not the way controls were selected in the case-control study on PM, and it is therefore difficult to directly compare odds ratio estimates obtained from ULR and CLR, and hazard ratio estimates obtained from WC1 and WC2.

## Discussion

Our simulation results suggest that the superpopulation variance estimator [13] provides adequate coverage probabilities of the CI when using the time-dependent weights proposed in the WC model to estimate the effect of time-varying exposures in case-control studies. Indeed, our simulation results shows much better coverage probabilities of the CI resulting from the superpopulation estimator than those resulting from the robust variance estimator. However, our application to PM suggests that the two variance estimators give similar 95% CI when the disease is very rare. This is consistent with the results of Lin [13] who showed that the use of finite-population variance estimator (i.e. robust variance) results in reasonable coverage probabilities if the inclusion probabilities are low, but poor coverage probabilities if the inclusion probabilities are high. It should be noted that both robust and superpopulation variance estimators are easy to implement using most statistical softwares.

Our simulation results also confirmed that the WC model is an alternative method for estimating the effects of time-varying exposure variables in case-control studies. In particular, when compared to standard logistic regression that did not dynamically account for the different values of covariates over lifetime, the WC model tended to provide more accurate estimates of the effects of variables for which an important percentage of subjects had time-varying values over lifetime, such as intensity. However, the superiority of the WC did not persist when more than one control were selected from the risk set. Our results also suggest that the estimates from the WC model are not more affected by correlations between time-dependent covariates included in the model than logistic regression with fixed-in-time covariates. Note that the modelling of the exposure in the WC model could further be improved by incorporating some more complex function of the trajectory of the exposure over time that have recently been proposed [28-30].

The application of the WC model requires estimating the age-conditional probabilities in the source population for population-based case-control studies, or in the full cohort for nested case-control studies. In our application to population-based case-control data on PM, these probabilities were estimated from health statistics on the general French male population. Yet, our analyses were restricted to ever exposed males only who have much higher probability to develop PM than the general French male population. Further studies are needed to investigate the impact of biased estimates of the age-conditional probabilities on the WC estimates. Accounting for uncertainty in the weight estimates could further improve the variance estimator [31]. In addition, controls in our case-control data set on PM were frequency matched to cases on birth year. To account for this stratification variable in the design, we included it as a covariate in the WC models. However, it would be interesting to consider accounting for this frequency matching variable in the weights of the WC models [12], and to investigate the performance of the resulting estimators through simulation of frequency matched case-control data. This would be all the more important that frequency matching is largely used in population-based case-control studies. It should also be mentioned that depending on the controls selection strategy, hazard ratio estimates from the WC model may not measure the same quantity as odds ratio estimates from the logistic regression. While the hazard ratio from the WC model estimates a rate ratio, the odds ratio may estimate another quantity depending on the control selection strategy [24].

The WC model with time-dependent variables requires also information on the values of the covariates at each event time, so at each age of diagnosis in cases. Such information may be missing, and different approaches could be considered to impute these values. However, further studies are needed to assess the impact of measurement errors of the time-dependent covariate values. Indeed, missmodeling the covariates has already been shown to induce bias in sandwich variance estimator based on dfbetas of unweighted Cox model for nested case-control analysis [32]. A variance estimator based on Schoenfeld residuals provided better variance estimates for severe model misspecification [32]. It may be of interest to further investigate such an estimator for misspecified time-dependent covariates in the WC model. Some further joint modelling between the WC model and the time-dependent covariate process could also be investigated as an alternative, especially for internal time-dependent exposure variables [33]. However, in most case-control studies on occupational exposures, the occupational history is sufficiently well investigated to allow the elaboration of quite accurate time-dependent covariates, as in our application on asbestos and PM.

## Conclusion

We believe that the WC model using the superpopulation variance estimator may provide a potential alternative analytical method for case-control analyses with detailed information on the history of the exposure of interest, especially if a large part of the subjects has a time-varying exposure intensity over lifetime, and if only one control is available for each case.

## Abbreviations

ASE: Average standard errors; CI: Confidence interval; CLR: Conditional logistic regression; JEM: Job-exposure matrix; MIE: Mean index of exposure; PM: Pleural mesothelioma; RMSE: Root mean squared error; SDE: Standard deviation of the estimates; ULR: Unconditional logistic regression; WC: Weighted Cox model.

## Competing interests

The authors declare that they have no competing interests.

## Authors’ contributions

HG has drafted the manuscript, programmed and run the simulation study, analyzed the case-control data on mesothelioma, and has contributed to the interpretation of all the results. AL has provided the case-control data on mesothelioma and has revised the manuscript. KL has drafted and revised the manuscript, has designed the simulation study, and supervised HG in all stages. All authors read and approved the final manuscript.

## Pre-publication history

The pre-publication history for this paper can be accessed here:

http://www.biomedcentral.com/1471-2288/13/18/prepub
